# Development, Implementation, and Process Evaluation of a Theory-Based Nutrition Education Programme for Adults Living With HIV in Abeokuta, Nigeria

**DOI:** 10.3389/fpubh.2019.00030

**Published:** 2019-03-12

**Authors:** Temitope K. Bello, Gerda J. Gericke, Una E. MacIntyre

**Affiliations:** Department of Human Nutrition, Faculty of Health Sciences, University of Pretoria, Pretoria, South Africa

**Keywords:** HIV, behavioral theories, nutrition education, quality of life, nutrition education resources, program development, nutrition

## Abstract

**Introduction:** Healthy diets play a role in the management and care for adults living with HIV/AIDS (ALH). Appropriate nutrition education (NE) is necessary to equip ALH with relevant knowledge and skills for healthy eating. A needs assessment identified the need for a nutrition education programme (NEP) as part of the nutrition service for ALH in Abeokuta, Nigeria. The aim of this study was to design a theory-based NEP and to evaluate the implementation process among ALH attending selected federal and state hospitals in Abeokuta as out-patients.

**Materials and Methods:** An exploratory descriptive needs assessment in the qualitative and quantitative domains was conducted among a convenient sample of ALH (*N* = 243) at the selected hospitals. The quantitative needs assessment identified needs for improvement in the primary outcome [quality of life (QoL)] and the secondary outcomes [quality of dietary intake, nutrition knowledge, attitudes, and practice (KAP), and anthropometric status]. Participants' perceptions of the NEP were obtained using an interviewer administered questionnaire among 70 ALH who attended the implementation of the NEP and process evaluation thereof at the intervention hospital.

**Results:** The qualitative results identified a lack of knowledge on planning varied meals with limited resources. The identified needs, existing guidelines and literature were integrated with appropriate constructs of the Social Cognitive Theory (SCT) and the Health Belief Model (HBM) into the NEP. The NE manual, participant's work book, flipcharts, and the brochure were tailored to address the identified challenges.

**Discussion:** The process evaluation showed that the NEP was implemented as planned and that the participants' perceptions were positive. The use of the NE manual, participant's work book, flipcharts, and brochure demonstrated the practicality of incorporating behavioral theories in NE for ALH.

## Introduction

A healthy diet should be part of the management of adults living with HIV/AIDS (ALH). Nutrition education (NE) has been found to improve the quality of life (QoL) and general well-being of ALH (1–3). The World Health Organization (WHO) (4) and American Dietetic Association (ADA) (5) in their resolution have urged nutrition and dietetics experts globally to give thoughtful attention to the NE of ALH to improve their health status (4). Research evidence has shown that antiretroviral therapy (ART) and micronutrient and macronutrient supplements alone, without nutrition education (NE), cannot reduce morbidity and mortality rates associated with HIV-infections (3).

Nutrition behaviors of people cannot be easily changed without identification of the mediators of their behavior (6). Appropriate alignment of an intervention to address the mediators of behavior can enhance intervention efficacy (7, 8). The constructs of learning theories in behavior based education have revealed success not only for fruit and vegetable consumption (9), but also for HIV prevention, smoking cessation (10), and other health behaviors. Behavioral theories are valuable in informing the development and implementation of a nutrition education programme (NEP) and in providing an understanding of the basis for the success or failure of interventions (6, 8, 11).

There seems to be no recent literature reporting on the development and implementation of a NEP for ALH to improve QoL, nutritional knowledge, individual dietary diversity scores (IDDS), and anthropometric status. In health behavioral education research, the Social Cognitive Theory (SCT), Health Belief Model (HBM), Social Network or Support Theory, Theory of Planned Behavior or Reasoned Actions (TPB or TRA) and Trans Theoretical Model or Stages of change (TTM or SC) are the most established and commonly used theories (12, 13).

The joint United Nations Programme on HIV/AIDS (UNAIDS) reports confirmed that the mortality rate related to HIV and AIDS diseases is high in developing countries such as Nigeria where application of an existing national guideline on nutritional care and support for people living with HIV/AIDS (PLWHA) seems to be inadequate (14). Hence, NE aimed at providing ALH with adequate nutrition knowledge needed for healthy dietary practices and improved QoL should be considered (3, 15). The aim of this study was to design a theory-based NEP and to evaluate the implementation process among the ALH attending selected federal and state hospitals in Abeokuta.

## Needs Assessment for the Development of the Nutrition Education Programme

### Study Design

A cross sectional exploratory and descriptive study design in the quantitative and qualitative domains was used for the needs assessment.

### Population and Sampling

Convenience sampling was used to recruit 243 males and females (18–50 years; on ART for ≥ 3 months but ≤ 5 years) from the selected hospitals out-patient departments after obtaining ethical approval from Faculty of Health Science Research Ethics Committee, University of Pretoria (South Africa), and the two selected hospitals in Abeokuta.

### Data Collection

Previously validated questionnaires were used to obtain quantitative data in respect of QoL (16), nutrition KAP (17–19) and dietary quality using the IDDS (20). Anthropometric status (body mass index (BMI), triceps skinfolds and mid-upper arm circumference) was measured using standard techniques (21, 22). Data collection instruments were translated into the local language, Yoruba, according to recommended procedures (23).

Four focus group discussions (FGDs) (20 participants) were held until data saturation was reached to explore participants' dietary patterns.

### Data Analysis

Descriptive statistics were used to describe the participants' demographic data. The elements (construct/subscales) of QoL, KAP, IDDS, and anthropometric status were summarized by sex using frequencies. Stata statistical software (release 10, 2007) was used for all quantitative analyses. The thematic framework approach was used to analyse the qualitative data (24).

### Challenges Identified by the Needs Assessment

The need for the development of the NEP to improve the QoL, nutrition KAP, IDDS, and anthropometric status of ALH was identified (Table 1). The quantitative results were complemented by the FGDs results.

**Table 1:** Summary of participants' needs identified from the needs assessment.

**Variable**	**N**	**Results**	**Needs identified**
QoL	169	Mean scores in physical functioning (47%), role limitation due to physical health (46%) and role limitation due to emotion (56%) cannot be considered as good (16, 19)	QoL was poor
Nutrition knowledge	235	Mean scores for knowledge: meal planning (33%), food preparation (54%), food purchasing (50%), and general nutrition knowledge (49%)	Poor nutrition knowledge and need for NE
Dietary diversity	206	Overall dietary diversity of the participants was poor; 134 (65%) had IDDS of ≤ 4. Food groups not consumed by 42% or more of participants: dark green vegetables, (42%) vitamin A rich fruits and vegetable (42%), other fruits and vegetables (62%), legumes and nuts (49%) organ meats (100%), milk and milk products (79%), and eggs and egg products (74%)	Unvaried diets and food insecurity
Anthropometric status	228	Mean BMI (24 kg/m^2)^, MUAC (for wasting < 22cm for female and < 23 cm for male) and TSF (31.3 mm) were within the range associated with good health, but 22% were overweight, 14% obese and 8% underweight	Guidance to maintain anthropometric status associated with good health
Focus group discussions (FGDs)	20	Financial constraints and cost of suitable foods to be their major challenges to food intake. Junk food outlets were regarded as suitable places to find healthy food	NE on meal planning, dealing with barriers to healthy eating, food budget, and purchasing

*QoL, Quality of life; KAP, Knowledge, attitudes and practices; BMI, Body mass index; MUAC, Mid upper arm circumference; IDDS, Individual' dietary diversity score*.

## Development of the Nutrition Education Programme

The development process was guided by the steps for designing a theory-based NEP as described by Sahyoun et al. (25) and Contento (26). The nutrition education content and materials are summarized in Tables 2, 3.

**Table 2:** Nutrition education programme content.

**Week**	**Lesson**	**Content description**
1	Understanding HIV/AIDS and the impact on health and nutritional status	• What is HIV and AIDS? • What is the immune system? • What are the symptoms of HIV/AIDS? • What are the modes of transmission of HIV? • What is the impact of HIV/AIDS on health and nutritional status?
2	Management of HIV/AIDS	• Drug therapy including the interaction with nutrients • Dietary management and nutritional care in HIV/AIDS management
3	Guidelines on good nutrition: well-planned varied meals	• Good nutrition and well-planned varied meals based on classes of food • Importance of good nutrition
4	Guidelines on good nutrition: well-planned varied meals continued	• Nigerian guidelines on nutritional care and support for people living with HIV/AIDS
5	Good nutrition: Planning well planned varied meals continued	• Group planning of well-planned varied meals based on available foods and resources
6	Good nutrition: management of nutrition related problems during HIV infections	• Nutritional problems and how to deal with them: • Weight loss • Diarrhea • Fever • Anemia
7	Tips for positive living	• Tips for positive living (medication and foods). • Food handling: animal products, fruits and vegetables • General hygiene • Importance of exercise/physical activities for positive living in HIV/AIDS
8	Quality of life and HIV	• What is QoL? • Who is responsible for your QoL? • How to improve your QoL?
9	Dealing with barriers to healthy eating	• Facilitate group discussion on overcoming barriers to healthy eating
10	Management of food budget and purchasing	• Describe the purpose of meal planning • Describe tips for shopping • Describe how to use limited budgets for healthy foods
11	Revision	
12	Question time and evaluation	Obtain participants feedback on: • Experience with training components • Experience with the duration of education programme • Perceptions on overall NEP delivery quality and benefits

*AIDS, Acquired immunodeficiency syndrome; SCT, Social cognitive theory; HBM, Health belief model; HIV, Human immunodeficiency syndrome; NEP, Nutrition education programme; QoL, Quality of life; TB, Tuberculosis*.

**Table 3:** Theory-based strategies and practical applications in educational activities.

**Strategies**	**Theoretical construct used**	**Application**
Use of flipcharts during the training session	HBM - Cue to action - Perceived severity	• Encourage participants to act on cues to healthy behavior • Connect participants to reality by showing pictures on the flipcharts • Give practical insights on the possible risk factors associated with HIV/AIDS such as: wasting, TB, diabetes, cardiovascular diseases
	SCT - Behavioral capability	• Provide behavioral capability through provision of sufficient information needed to apply knowledge
Use of work book	HBM - Perceived barriers	• Assist participants to acquire skills and knowledge to overcome emotional and financial barriers
	SCT - Self-efficacy - Behavioral capability - Outcome expectation	• Build self-confidence when participants respond correctly to questions • Initiate group interaction during the education sessions to give room for involvement of peers • Encourage participants to discuss challenges and how to overcome them • Develop participants' skills to plan varied meals with limited resources
Use of trainer's manual for one on one teaching	HBM - Cue to action - Perceived barriers	• Engage participants in one on one discussions to help them to identify healthy and unhealthy dietary behaviors • Expose individuals to cue for good adherence and reasons for poor adherence
	SCT- Self-efficacy - Behavioral capability - Outcome expectation	• Facilitate group discussions to enhance acquisition of knowledge • Write on the board and show flipchart pictures to facilitate participants' acquisition of knowledge
Assessment of participants' prior knowledge and revision of previous lessons	SCT - Self-efficacy - Behavioral capability	• Participants' feedback to improve their self-confidence in performing acquired skills and knowledge • Encourages participants to demonstrate the knowledge they acquired through response

*HIV/AIDS, HIV-Human immunodeficiency syndrome; AIDS, Acquired immunodeficiency syndrome; SCT, Social cognitive theory; HBM, Health belief model; TB, Tuberculosis*.

The trainer's manual and participant's work book were designed to cover topics addressing the problems identified during the needs assessment.

The flipcharts consisted of 15 charts adapted from existing charts (27–30) and were used to create visual connections, provide cues to action and motivate participants to adopt healthier behaviors (Table 3).

The brochure summarized the manual by covering all the problems identified in the needs assessment (e.g., impact of HIV/AIDS on health and the nutritional status of the people living with HIV/AIDS and dietary management of HIV).

## Implementation and Process Evaluation of Nutrition Education Programme

### Study Design

The implementation was part of a quasi-experimental design in which one of the two sites was randomly allocated to the implementation to evaluate the impact of the NEP on QoL, nutrition KAP, IDDS, and anthropometric status. Participants at the intervention site received face to face group teaching of the NEP with the education materials over 12 weeks. The process was evaluated at the end of week 12.

### Population and Sample

The target population comprised ALH attending the study sites as out patients. The overall mean score for the QoL scale for the needs assessment was 65% (±14.7) indicating that a sample of at least 50 participants would have 90% power to detect an improvement of 10% in the QoL construct scores of the intervention group (*p* = 0.05). A drop out of 50% was considered for the intervention group; therefore a sample of at least 100 participants per group was appropriate.

The participants were stratified according to the duration on ART (0–24 months > 24 months) and sex during recruitment. Stratification for sex was based on the results of the needs assessment (25% males: 75% females) to ensure that the sample was representative of the population sex distribution. The stratification of participants according to the duration on ART was to control for the confounding factor of ART.

### Implementation

Intervention participants were allocated on a first come first served basis to groups of 13–15 participants per group. Each group met once a week for 12 weeks, and 60 minutes per session was planned and achieved.

The researcher (TKB), a registered nutritionist, facilitated most of the NE sessions with the assistance of trained research assistants. The NE sessions were conducted between Monday and Friday.

A separate venue, from where the participants received ART, was secured for the implementation. The intervention and control participants were given a brochure at the point of recruitment.

### Process Evaluation

A questionnaire containing eight open ended questions developed by the researcher was administered to the intervention group at week 12 to obtain the participants' perceptions of the programme in terms of the reach, fidelity, length of the education sessions and whether personal expectations were met (26, 31).

### Data Analysis

The quantitative data were analyzed using descriptive statistics (percentages and frequencies). The intervention participants' responses to the opened ended questions were analyzed using the thematic framework method of analysis (24, 32, 33). The main themes that emerged were discussed in categories which included participants' perceptions of the NEP.

## Results

A total of 100 participants was allocated to seven NE groups. Table 4 shows the NE attendance rate (reach) during the education sessions over 12 weeks. The reasons for not achieving 100% attendance included death, bereavement, accident, no longer interested, work obligations, relocation to other areas, no response to reminder calls and family challenges.

**Table 4:** Summary of the intervention group attendance rate during the implementation of the nutrition education programme.

	***n***	**Attendance of ≥ 50 % of the weekly education sessions *n* (%)**	**Drop-out *n* (%)**
Group 1	15	11 (73.3)	4 (26.7)
Group 2	15	12 (80)	3 (20)
Group 3	15	11 (73.3)	4 (26.7)
Group 4	15	12 (80)	3 (20)
Group 5	13	11 (84.6)	2 (15.4)
Group 6	14	8 (57.1)	6 (42.9)
Group 7	13	12 (92.3)	1 (7.7)
Total	100	77	23

*n, number; drop-out, participants not available at week 12 assessments*.

Tables 5 summarizes the perceptions of the participants on the impact and presentation of the NEP on their knowledge and behavior.

**Table 5:** Summary of the perceptions of the participants on the impact and presentation of the nutrition education programme.

**Theme**	**Sub-theme**	**Participants quotes**
**IMPACT**		
Participants' expectations	Participants' personal expectations being met	“*Yes the training met my expectations because I learnt new things”*
New knowledge	New things learnt during the programme	“*Yes I learn something new”*
New knowledge that participants anticipate implementing	Nutrition recommendations and medication side effects	“*That we should not add extra salt to our foods after preparation” “I learnt that I should first take my medication before eating” “I learnt that patients on Efavirenz should avoid too much of starchy foods”*
	Strategies for medication adherence	“*I learnt that I should take my medication at the right time” “I learnt that we should not smoke cigarette” “I learnt that I should not miss my clinical appointment” “I learnt that I should not drink alcohol”*
	Quality of life	“*I learnt that my quality of life can be improved by accepting that challenges of life are to help me grow” “I learnt that whatever I eat can affect my emotions (moods)” “I learnt that I can live long by eating varieties, taking medications and worrying less” “I learnt personal hygiene can help me improve my quality of life”*
	Benefits of eating variety of food	“*I learnt that eating varieties can enhance nutrient adequacy” “We learnt that we should eat different varieties of foods because they will make our body to function well” “I learnt that I don't need to be rich to eat varieties, even with my limited resources I can still eat varieties” “I learnt that in planning my daily menu, fruits should be given daily consideration” “I learnt we should eat varieties of foods during pregnancy”*
	Tips on planning meal on a limited budget	“*It pays to plan our meals and make budget for food purchase”*
	Tips for positive living	*“I learnt about good ways of living with my status such as personal hygiene” “I learnt that to stay healthy, I need regular exercise”*
	Tips for shopping	“*I learnt that I should buy healthy foods”*
Overall impact of the education sessions	Impact of medications on health status	“*I have learnt to use my medication every day”*
	Take home messages	“*I learnt that too much of fatty foods should be avoided” “I learnt that we should not eat food that contain lots of fats” “Budget making” “I have learnt how to plan varieties of meals with limited resources” “I learnt that street foods are not the best for me” “I learnt about eating right”*
**PRESENTATION**		
Understanding of the NEP	Adequate understanding of the NEP	“*Yes”*
Learning challenges during the education sessions	Needs more education sessions	“*None” “I understood everything”*
Length of the education session	Adequacy of the duration of education sessions	“*The time was ok” “I want the duration to be extended” “The time was well managed” “The time was too short for the education sessions”*
Recommendations	General comments	“*The education session should continue” “The facilitator really came down to the level of the participants” “The education sessions were good and useful to us” “The teaching sessions were interesting and informative”*

*NEP, Nutrition education programme*.

## Discussion

The participants' retention rate was 77% (in attendance for ≥ 50% of the weekly education sessions) at week 12. This is higher than the results reported by Hatsu et al. (34) and Keithley et al. (35). Hatsu et al. (34) observed that 57% of the intervention participants attended more than 50% of the education sessions. Keithley et al. (35) reported that 52% of the intervention group was available for the week 24 assessment in a 12 month randomized control trial among 90 ALH in Chicago. The high retention rates in our study could be explained in terms of enthusiasm and appreciation of the participants. The fact that participants might not have received any nutrition education materials related to HIV-infections prior to the present study might be a motivational factor for good attendance rates. Fortnightly telephonic reminders and encouragement given to participants to attend the next hospital appointment and nutrition education session could also have contributed.

The process evaluation showed that the programme delivery rate was 100% as had been planned. This result is in agreement with a study among ALH in the US that reported 100% NEP delivery rates (34). In that study each of the four nutrition education sessions was delivered by a registered nutritionist for 45–60 min on the same day to two groups with 15 participants per group (34).

The NEP was based on the identified needs (Table 1). Hence, various topics such as guidelines on good nutrition via well-planned varied meals and tips for positive living were part of the lessons (Table 2). The information on varied meals and strategies for medication adherence could be responsible for the desirable response from the participants that they learnt that planning meals containing a variety of foods is not necessarily expensive and daily intake of their medication is advantageous to their health status. Therefore, identification of the participants needs and addressing them can lead to positive perceptions toward behavioral change (12, 13, 36).

Perceived severity is a construct of the HBM, understood as the possible perception that individuals have about their health status (37), and based on the beliefs and knowledge of an individual (37). The NEP informed participants of the consequences of eating unvaried diets such as weight loss, overweight, anemia, fatigue, and poor immunity. Flipchart pictures were used to contextualize the reality of the severity of the virus. ALH were informed of a study in the United States that reported that adherence to ART enhanced long term viral suppression and increase survival (38). The participant's belief that adherence to ART could delay and reduce the severity of HIV disease progression could promote positive behavior change.

A perceived barrier explains an individual's perception about the obstacles to adopting a new behavior (39). Participants' direct quotes showed that participants perceived barriers such as cost of healthy food were well addressed: “*even with my limited resources varieties of foods could still be consumed*.” Adherence rates can be improved when barriers are addressed (38). The HBM perceived barrier construct was addressed by teaching participants on how to deal with barriers to healthy eating and sharing tips on food budgeting and purchasing.

Cues to action are events, people or things that can make people change their behavior (39), e.g., brochures, teaching, media reports, advice, and group interactions (36). A nutrition brochure, the participant's work book and flipcharts were used to provide cues to action. Participants had the opportunity to interact with peers and the facilitator of the NEP for nutrition guidance. Participants' direct quotes on tips for positive living, strategies for medication adherence, and tips for shopping and meal planning revealed that the education materials provided cues to action which could lead to behavior change (Table 5).

Behavioral capability entails knowledge and skills required to perform appropriate behaviors (40). In this study, it was expected that participants would acquire the nutrition knowledge and attitudes needed to embrace behavioral capability. The strategy of reinforcing information through the use of the NE materials was applied (41). Participants' direct quotes that they learnt planning varieties of meals even with limited resources revealed behavioral capability (Table 5).

A perceived benefit is a personal opinion or an individual's conclusion on a new behavior and can lead to adoption of healthier behaviors (37). The NE materials emphasized that varied foods are needed for proper functioning of the immune system and good QoL. Participants' responses on take home messages such as regular exercise, avoidance of fatty foods and eating of varieties on a daily basis showed that participants acquired knowledge based on perceived benefits (Table 5).

Self-efficacy is an individual's boldness or self-confidence to perform newly acquired skills (42). This construct of the SCT was facilitated by engaging participants in revision of previous lessons with inputs from the participants. Participants were also engaged in role playing on planning a healthy meal with limited resources. Participants were asked questions such as descriptions of foods available in their environment, tips for positive living, and prior knowledge of management of HIV/AIDS in order to enhance their self-confidence. The self-efficacy construct was successfully used in an 8 week community based NEP for 40 low-income adults in Seattle to improve fruit and vegetable consumption (43).

The participants in this study were expected to have positive change of attitudes by performing appropriate nutrition actions in order to improve QoL. The outcome expectation construct was incorporated into the NE materials through an explicit explanation on the benefits of good nutrition during HIV-infections. Participants' direct quotes that the education sessions were informative and timely could be responsible for the positive responses of knowledge acquisition (Table 5). Participants' perceptions of the benefits of good nutrition could enhance their readiness to adopt a healthy nutrition lifestyle (44).

Incorporation of the selected constructs of the SCT and HBM in the development of the NE materials could be responsible for the acquisition of new knowledge. Another NEP used the SCT to improve fruit and vegetable intakes among 754 out-patients in rural Virginia (45). The study indicated significant improvement in knowledge of fruit and vegetable consumption among the intervention group (45). Heim et al. (46) also implemented a 12 weeks garden based NEP that was planned by using the constructs of the SCT and experiential learning theories among children with low fruit and vegetable intakes in the US, leading to significant increase in their fruits and vegetables intake (46).

The participants' perceptions of the presentation of the programme showed that the education sessions were informative and interesting. Several factors may have contributed to these positive responses. Experts have confirmed that the facilitator, the mode and the format of NE delivery play a vital role in the effectiveness of the implementation of a NEP (26). The use of group education which was reported to be easier, cheaper and to require less skill or professionalism than individual counseling (26) could also have contributed to the acquisition of knowledge.

## Conclusion

The results of the needs assessment informed the necessity for the development of the NEP. The use of well-planned education content and materials (flipcharts, brochure and participant's work book) and teaching of NE by a registered nutritionist could be responsible for the positive feedback received in the present study.

## Ethics Statement

Ethical permission for the study was sought from the Faculty of Health Sciences Research Ethics Committee, University of Pretoria, South Africa (number: 311/2014) and from the two hospitals (Hospital 1: FMCA/470/HREC/2014; Hospital 2: SHA.144/vol. II/97). Permission to access the participants' medical records was obtained from the Chief Executive Officers (CEO) of the two hospitals. No participant was allowed to participate without understanding and signing the informed consent for the study in a manner conforming with the declaration of Helsinki. Confidentiality and freedom to withdraw from the study at any time without prejudice were assured.

## Author Contributions

TB, GG, and UM were involved in the conception and research design of the study. TB supervised recruitment of participants and data collection. GG and UM participated in the data analysis. All authors were involved in the interpretation of the data and the writing of the of the paper. All authors approved the final version of the paper.

### Conflict of Interest Statement

The authors declare that the research was conducted in the absence of any commercial or financial relationships that could be construed as a potential conflict of interest.
